# ggalign: Bridging the Grammar of Graphics and Biological Multilayered Complexity

**DOI:** 10.1002/advs.202507799

**Published:** 2025-09-15

**Authors:** Yun Peng, Shan Jiang, Yuxuan Song, Peng Luo, Jianfeng Li, Dehua Hu, Jian‐Guo Zhou, Guangchuang Yu, Tao Xu, Shixiang Wang

**Affiliations:** ^1^ Department of Urology Peking University People's hospital Beijing 100044 China; ^2^ Department of Biomedical Informatics School of Life Sciences Central South University Changsha 410013 China; ^3^ Department of Oncology Zhujiang Hospital Southern Medical University Guangzhou 510282 China; ^4^ State Key Laboratory of Medical Genomics Shanghai Institute of Hematology National Research Center for Translational Medicine Rui‐Jin Hospital School of Medicine Shanghai Jiao Tong University Shanghai 200025 China; ^5^ Department of Oncology The Second Affiliated Hospital of Zunyi Medical University Zunyi 563006 China; ^6^ Department of Bioinformatics School of Basic Medical Sciences Southern Medical University Guangzhou 510515 China

**Keywords:** composable visualization, data visualization, ggalign, grammar of graphics, omics

## Abstract

Data visualization is essential for exploring and communicating complex biological datasets. As omics data grow in scale and complexity, there is an increasing demand for visualization tools that are both flexible and extensible. We present ggalign, an R package that extends the ggplot2 ecosystem by introducing an integrative framework for composable visualization. Designed to overcome limitations of existing tools, ggalign supports modular, data‐aware layouts‐including circular, stacked, and quadrant‐based configurations‐and enables the representation of diverse data relationships, such as one‐to‐many and many‐to‐one connections. Its ability to reorder and group observations using data‐driven or domain‐specific criteria enhances the interpretability of high‐dimensional datasets. Moreover, ggalign introduces a novel linking mechanism for visualizing interconnections across heterogeneous data types, as demonstrated in genomic and microbiome case studies. Together, these features position ggalign as a versatile and reproducible solution for multi‐omics research, supporting both exploratory data analysis and publication‐ready presentation.

## Graphical abstract

The ggalign package provides a comprehensive framework for composable visualization built on top of ggplot2. Its primary purpose is to enable the construction of complex multi‐plot layouts with consistent observation alignment across plots, particularly for high‐dimensional data contexts such as genomics, transcriptomics, and microbiome studies.

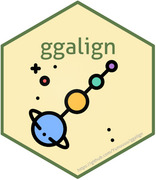

## 1 Introduction

Data visualization is fundamental to modern data analysis, serving as a critical medium for exploring, interpreting, and communicating complex datasets. In biomedical research, the increasing availability of multi‐dimensional data‐spanning genomics, transcriptomics, epigenomics, microbiomics, and beyond poses significant challenges for effective visualization.^[^ ^1^, ^2^, ^3^, ^4^, ^5^, ^6^ ^]^ These datasets often encode intricate, multi‐layered relationships that require composable, flexible visualization strategies capable of capturing both structure and nuance.

Composable visualizations are particularly valuable in this context, as they enable the dynamic arrangement of diverse visual elements, supporting multi‐scale comparisons and revealing subtle associations across biological hierarchies. However, existing solutions for composable visualization remain limited in scope or generality. Domain‐specific tools such as ComplexHeatmap^[^ ^7^ ^]^ and ggtree^[^ ^8^ ^]^ are tailored to heatmaps and phylogenies, respectively. Meanwhile, frameworks like aplot^[^ ^9^ ^]^ and Marsilea^[^ ^10^ ^]^ support alignment of plots but are restricted to simple one‐to‐one mappings, rendering them suboptimal for complex data structures that involve one‐to‐many or many‐to‐one relationships for example, mapping microbial species across taxonomic ranks or associating gene sets with functional pathways.

Here we introduce ggalign, an integrative framework for composable visualization that extends the grammar of graphics^[^ ^11^ ^]^ to support expressive and customizable alignment of multi‐panel plots. Built on the ggplot2^[^ ^12^ ^]^ ecosystem, ggalign provides a unified abstraction for constructing and aligning diverse graphical objects across complex relational structures. Its design allows researchers to visualize interconnected omics layers in a cohesive, publication‐ready format without sacrificing customizability or reproducibility.

In this article, we describe the conceptual framework, implementation, and key features of ggalign. We demonstrate its utility across a range of biomedical applications, including pan‐cancer microbiome profiling, integrative genomic analysis, and single‐cell transcriptomics, highlighting how it facilitates deeper insight and clearer scientific communication. By bridging the gap between aesthetics and analytic precision, ggalign empowers researchers to construct visual narratives that scale with the complexity of modern data.

## 2 Results

### 2.1 Integrative Framework for Composable Visualization

 ggalign is a grammar‐based framework for composing multi‐panel visualizations in R. It extends the expressive capabilities of ggplot2 through a dual system of data‐free and data‐aware composition. The core architecture spatially arranges plots using explicit layout logic, while optionally preserving consistent alignment of shared observations‐making it particularly suitable for complex multi‐modal visualizations in genomics and other data‐intensive fields.

#### 2.1.1 Data‐Free Composition

At its foundation is a plot composer architecture that spatially arranges independent plots‐an approach we refer to as data‐free composition, where layout is determined without assuming any data relationships. This paradigm is conceptually aligned with tools like patchwork or cowplot, but ggalign introduces significantly finer control over alignment, spacing, legend merging, and axis coordination. The central engine for data‐free composition is the *align_plots()* function, which accepts a mixture of plot types and graphic objects and arranges them into a structured layout with detailed control over positioning and guides (Figure S1A, Supporting Information).

Beneath *align_plots()* lies a modular transformation layer built on the generic *patch()* function and the Patch object system. The *patch()* function automatically converts a wide range of graphical inputs‐including ggplot2 objects, ComplexHeatmap, pheatmap, base R plots, and others‐into standardized grid‐compatible graphical objects (grobs) (Figure S1A, Supporting Information). The Patch object then encapsulates the layout semantics, alignment logic, and compositional behavior. This architecture makes the system highly extensible: users and developers can add support for new plot types by defining methods for *patch()* or by creating new Patch classes to implement custom layout strategies. This ensures seamless integration of novel graphical objects into complex multi‐panel layouts. To further support visual flexibility, ggalign includes modifier functions such as *free_align()*, *free_space()*, and *free_border()*, which allow selective relaxation of alignment constraints across plot components. This makes the system adaptable to a wide range of layout scenarios.

#### 2.1.2 Data‐Aware Composition

Building on the data‐free composition framework, data‐aware composition is the defining features of ggalign. It enables multiple plots that share common observational units‐such as genes or samples‐to be aligned such that each observation appears consistently across all panels. This ensures interpretability and coherence in complex, multi‐modal visualizations where preserving the correspondence of shared data points is critical.

Unlike data‐free composition, which arranges independent plots without enforcing data relationships, data‐aware composition imposes a strict constraint: observations must be precisely matched across all plots in a layout. This constraint enables integrated, multi‐panel visualization of high‐dimensional datasets by maintaining consistent ordering and grouping of observations.

**Figure 1:**
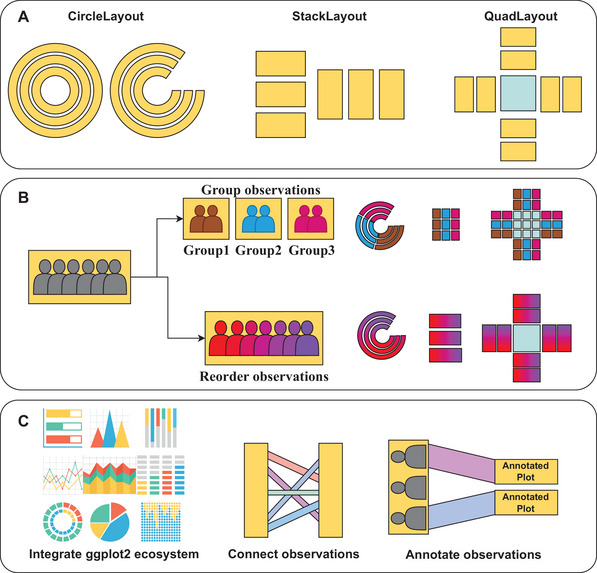
Overview of data‐aware composition features. A) Illustration of the three core layout systems implemented in ggalign: the CircleLayout organizes plots in a circular configuration, offering a compact view of interconnected relationships; the StackLayout organizes plots along a shared axis to facilitate comparison; the QuadLayout arranges four contextual annotations around a central reference to highlight interdependencies. B) Extensible and data‐aware methods for observation‐level grouping and reordering, enabling structure refinement and enhanced visibility of biologically meaningful patterns. C) Demonstration of observation‐level linking strategies, allowing visual integration and annotation of corresponding elements across plots.

The Layout S7 class system (Figure S1B; **Figure** **1**A, Supporting Information) underpins this capability by tracking axis domains, observation counts, and ensuring dimensional compatibility across plot components. It also manages user‐defined ordering and grouping to prevent misaligned visualizations, thereby preserving the identity of each observation throughout the multi‐panel layout.

Supporting this data‐aware framework is a sophisticated alignment system built around the CraftBox S7 class hierarchy, which serves as the user‐facing interface and wraps the underlying Craftsman R6‐like object system (Figure S1B, Supporting Information). The Craftsman encapsulates core logic for grouping, clustering, ordering, and adding plots to the layout, providing a comprehensive developer‐level toolkit for managing shared observations and their structural relationships.

Leveraging the Craftsman implementation, ggalign provides a suite of user‐friendly functions for organizing and reordering shared observations (Figure S1B; Figure 1B, Supporting Information). These include hierarchical clustering (*align_hclust()*), custom ordering (*align_order()*), k‐means clustering (*align_kmeans()*), and manual grouping (*align_group()*). When observations are reordered or grouped in one plot, identical transformations are applied consistently across all linked plots, maintaining global coherence as enforced by the Layout system. For visualization, ggalign provides functions such as *ggalign()* and *ggfree()* to add aligned or freely positioned plots to layouts, as well as *align_dendro()* for dendrogram alignment to visualize hierarchical relationships.

In addition, ggalign supports visual linking of observations via lines or polygons, implemented within the Craftsman system (Figure S1B; Figure 1C, Supporting Information). These connections are used in advanced tools like *ggmark()*, which annotate related observations across panels. The linking framework accommodates one‐to‐one, one‐to‐many, and many‐to‐many relationships, enabling expressive representations of complex biological structures such as gene modules, sample groupings, or interaction networks. It also facilitates the identification of coordinated or discordant patterns across omics layers. For example, as shown in Figure S2 (Supporting Information), genes such as *DCHS1*, *AKT3*, *SFRP1*, and *FHL1* in endometrial carcinoma display coordinated expression between transcriptomic and proteomic profiles, while others exhibit discordant behavior. To specify linked observations, users are required to utilize the formula syntax denoted by “∼”. A one‐sided formula links selected observations to an annotated plot, while a two‐sided formula (e.g., x ∼ y) explicitly defines links between observations across two distinct plots.

Complementing the Layout and CraftBox systems is the Scheme system, which defines visual or structural controls that apply to both Layout and CraftBox objects. Controls applied at the Layout level are inherited by associated CraftBox elements and applied to the plot during rendering. The Scheme system is explicitly designed for extensibility by developers. It provides a set of S7 generic functions—*scheme_init()*, *scheme_update()*, *scheme_inherit()*, and *plot_add_scheme()*—which can be overridden to define initialization, update, inheritance, and application behavior of new Scheme subclasses. Developers can extend this system to support new types of visual control, theming strategies, or layout logic, making the framework adaptable to a broad range of visualization scenarios. The package currently provides built‐in schemes for default theming (*scheme_theme()*), input data transformation (*scheme_data()*), and alignment specification (*scheme_align()*).

**Figure 2:**
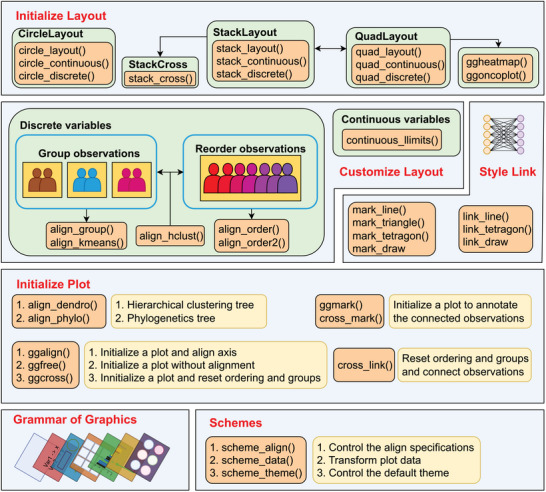
Overview of data‐aware composition functions.

This data‐aware paradigm is particularly powerful for genomics, transcriptomics, and other high‐dimensional biological datasets, where maintaining the correspondence between samples, genes, or other entities across multiple visualization modalities is crucial for interpretable analysis. By ensuring that the same biological entity appears at the same relative position across heatmaps, dendrograms, bar plots, and other visualizations, data‐aware composition enables researchers to trace patterns and relationships across different data views seamlessly. An overview of user‐facing functions supporting data‐aware composition is presented in **Figure** **2**.

### 2.2 Design Philosophy

 ggalign is built on a grammar‐based philosophy that extends the declarative syntax of ggplot2 to multi‐panel, observation‐aligned visualizations. It preserves ggplot2's familiar additive composition model (“+”), while introducing new semantics for alignment, coordination, and layout‐aware transformations (Figure S3, Supporting Information). This syntactic continuity ensures a minimal learning curve for users familiar with the tidyverse. Additionally, ggalign extends support for the “‐” and “&” operators, allowing elements to be applied across multiple plots efficiently.

The system treats aligned plots, layout containers, and control schemes as composable objects. Layouts function as plot‐like containers accepting geoms, annotations, and scheme specifications via the same additive syntax. This consistent interface design enables users to build complex visual structures incrementally‐inspecting, modifying, and extending layouts within a tidyverse‐compatible workflow.

Data‐aware plotting functions within ggalign are fully interoperable with ggplot2 geoms, stats, scales, and themes. Plots produced by ggalign can be further customized using the full range of ggplot2 syntax and semantics, allowing users to apply geoms, stats, and theming as they would with standalone ggplot2 plots.

The framework is also highly extensible for developers. Leveraging the R6‐like object system (ggproto) and S7‐based interfaces, developers can introduce new layouts, custom plot types, new layout behaviors, and additional control schemes while maintaining compatibility with the core grammar. For instance, we implemented a *circle_genomic()* function that extends *circle_layout()* to support circular visualizations of genomic data (Figure S4, Supporting Information), enabling the display of differentially methylated region enrichment in a compact, genome‐aware format.

By combining ggplot2‐style grammar with a robust infrastructure for plot coordination, ggalign delivers a unified interface that is both intuitive for users and extensible for developers, enabling coherent and scalable construction of complex, observation‐aligned visualizations.

Compared to existing composable visualization tools such as Marsilea,^[^ ^10^ ^]^ aplot,^[^ ^9^ ^]^ and domain‐specific platforms like ComplexHeatmap,^[^ ^7^ ^]^ ggalign offers a more general and unified solution. While other tools are typically restricted to fixed layout structures or single relationship types, ggalign enables the alignment and annotation of plots across a wide range of formats and analytical contexts (**Table** **1**; Table S1, Supporting Information). Its deep integration with the ggplot2 ecosystem ensures compatibility with established graphical grammars, allowing users to extend their visualizations with familiar aesthetic and statistical layers. This versatility makes ggalign particularly well‐suited for exploratory data analysis and integrative multi‐omics studies, where customized, data‐driven visualization is essential.

**Table 1:** Comparison with other composable visualization tools.

		ggalign	Marsilea^[^ ^10^ ^]^	Aplot^[^ ^9^ ^]^	ComplexHeatmap^[^ ^7^ ^]^
Language		R	Python	R	R
User Interface		Declarative	Declarative	Functional	Functional
Plot System		ggplot2 (Advanced plot system built on grid system)	Matplotlib	Build on patchwork which uses ggplot2	grid
Focus		General‐purpose composable visualization	Grid‐based composable visualization	Plot arrangement	Heatmap
StackLayout		√	√	√	√
QuadLayout		√	√	√	Heatmap only (discrete variables)
CircleLayout		√	×	×	×
Relationship	One‐to‐One	√	√	√	√
	One‐to‐Many/ Many‐to‐One	√	×	×	×
	Many‐to‐Many	√	×	×	×
	Crosswise	√	×	×	×
Annotate observations		√	×	×	√
Compatible with ggplot2		√	×	√	×

Rendering time in graphical systems typically scales with the number of visual elements. In statistical plots such as bar charts or boxplots, each graphical unit often summarizes multiple observations, resulting in relatively low rendering overhead. In contrast, plots with one graphical element per observation—such as heatmaps or scatter plots‐scale linearly with data size and can become computationally demanding. We benchmarked heatmap rendering performance across several commonly used R packages under increasing data sizes. ggalign demonstrates superior rendering speed compared to ComplexHeatmap (Figure S5A, Supporting Information). Additionally, our comparison of code complexity with ComplexHeatmap and ggplot2 indicates that ggalign has the lowest complexity among these packages (Figure S5B, Supporting Information) which contributes to greater maintainability and system stability.

**Figure 3:**
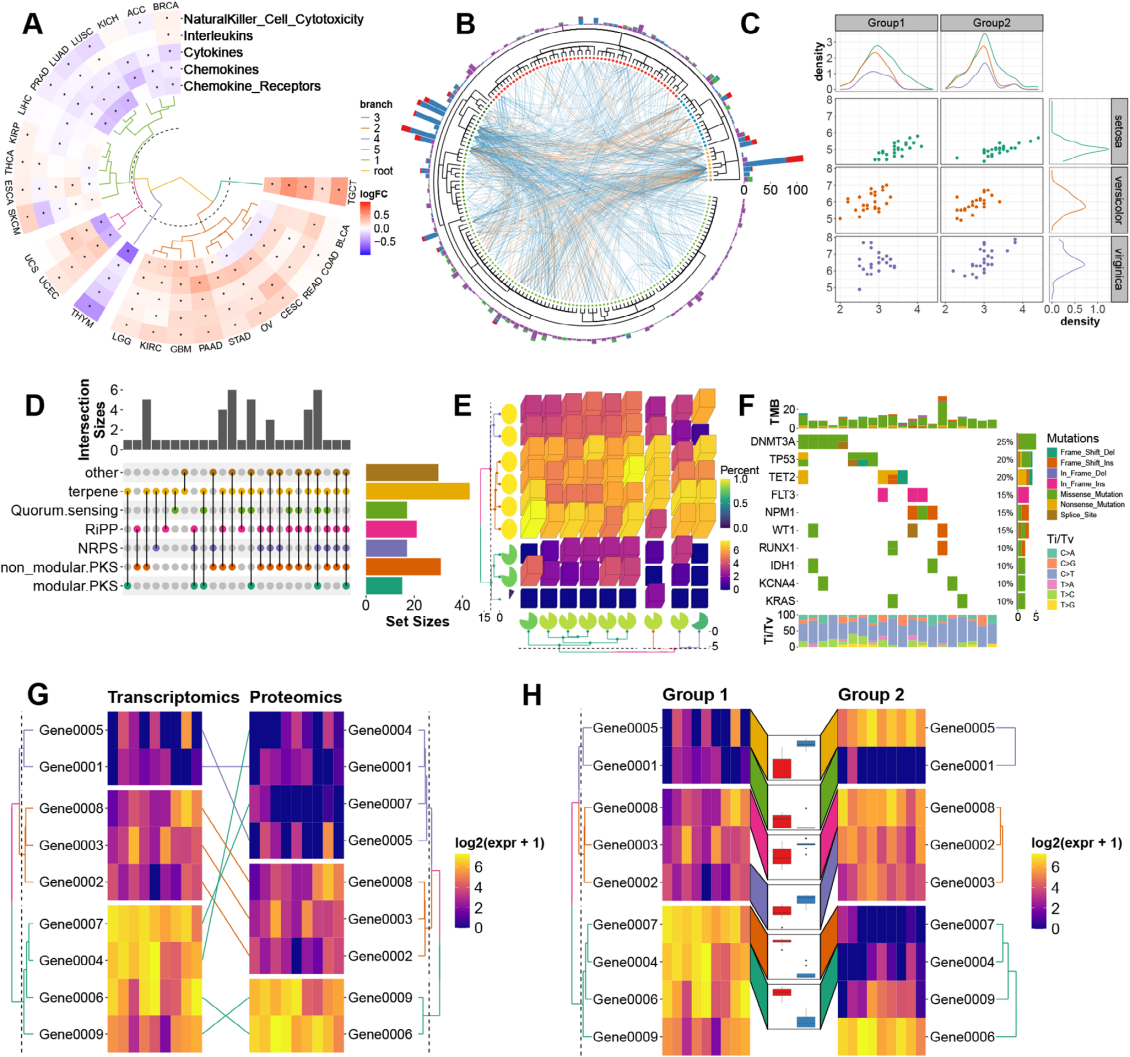
Overview of ggalign's visualization capabilities. A,B) Demonstration of CircleLayout. C) Demonstration of QuadLayout for continuous axes. D) Application of QuadLayout with an UpSet plot. E) Application of QuadLayout with a 3D Heatmap. F) Application of QuadLayout with an Oncoprint. G) Demonstration of StackLayout + QuadLayout with linked observations from two different orderings. H) Demonstration of StackLayout + QuadLayout with annotated plots for selected observations.

By combining a grammar‐based approach with layout coordination and observation alignment, ggalign bridges the gap between exploratory analysis and structured communication of scientific results. It enables researchers to construct visually coherent, analytically meaningful representations of complex data—advancing the visual language of modern biomedical research (**Figure** **3**).

### 2.3 Demonstrating ggalign's Functionality in Complex Data Visualization

We showcase ggalign functionalities by applying it to several published pan‐cancer datasets, including microbiome, single‐cell research, transcriptomics, and genomics research. The complete package reference is available at https://yunuuuu.github.io/ggalign/, with comprehensive documentation and tutorials at https://yunuuuu.github.io/ggalign‐book/, and a gallery of example figures at https://yunuuuu.github.io/ggalign‐gallery/.

#### 2.3.1 Case I: Intratumor Microbiome Characteristics in Different Tumor Types

To demonstrate the utility of ggalign for constructing interpretable, multi‐layered visualizations in complex biological datasets, we analyzed intratumor microbiome profiles across five tumor types from The Cancer Genome Atlas (TCGA)^[^ ^13^, ^14^ ^]^ project.

**Figure 4:**
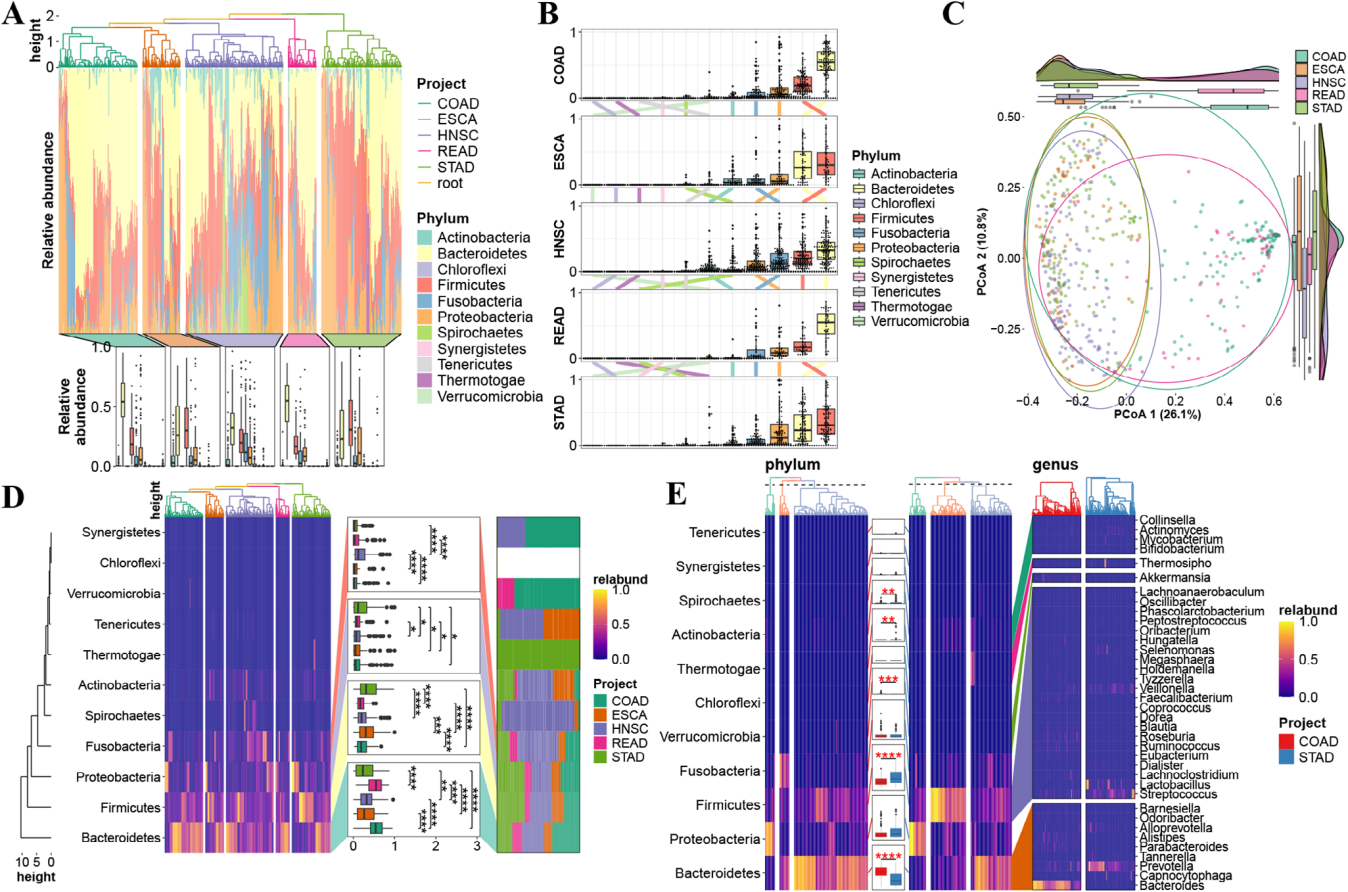
Exploration of intratumor microbiome characteristics among tumor types. A) Relative abundance of microbial phyla across five tumor types from TCGA, reordered by hierarchical clustering within each group. B) Transitions in phylum rank based on mean relative abundance across tumor types. C) PCoA plot along with boxplots and density plots for each dimension D) comparing the abundance of the top four phyla (“Bacteroidetes”, “Firmicutes”, “Proteobacteria”, and “Fusobacteria”) across all tumor types. *P*‐value was adjusted in each plot panel. E) Differential phyla and corresponding differential genera between COAD (colon adenocarcinoma) and STAD (stomach adenocarcinoma) are highlighted. *P*‐value was adjusted across all taxa. Label “*” means *P*‐value < 0.05, label “**” means *P*‐value < 0.01, “***” indicates *P*‐value < 0.001, and label “****” indicates *P*‐value < 0.0001.

We began by visualizing the relative abundance of microbial phyla across tumor types. Within each cohort, hierarchical clustering was used to reorder observations based on microbial composition. Using ggalign, we aligned annotated boxplots across groups while preserving this observation‐level reordering (**Figure** **4**A). In this context, the ggalign functioned as a data‐aware layout engine that maintained internal ordering while supporting visual comparability, enabling simultaneous inspection of within‐group variation and between‐group structure.

To explore cross‐tumor differences, we constructed a coordinated layout of boxplots linked by shifts in phylum abundance rankings (Figure 4B). Additional plots were incorporated to visually trace changes in relative abundance across tumor types, revealing the conserved dominance of Bacteroidetes, Firmicutes, Proteobacteria, and Fusobacteria,^[^ ^15^ ^]^ while clarifying the variability among less abundant phyla. ggalign's ability to align plots with differing observation orders enabled direct visualization of these transitions, enhancing the interpretability of taxonomic dynamics across tumors.

For a broader overview of group‐level variation, we performed principal coordinate analysis (PCoA) and used ggalign to enrich the main projection with marginal boxplots and density strips precisely aligned along each axis (Figure 4C). This modular yet unified layout enabled detailed inspection of sample distributions along each principal component while maintaining alignment with the central PCoA plot. The fine‐grained control over positioning and axis synchronization provided by ggalign ensured consistent scaling and cohesive interpretation.

To compare phylum‐level profiles in more detail, we next constructed a heatmap and an annotated boxplot focused on the four dominant phyla (Figure 4D). With ggalign, we embedded statistical annotations and maintained synchronized axes across panels, facilitating direct, side‐by‐side comparisons. This design emphasized the distinct phylum composition observed in stomach adenocarcinoma (STAD), particularly its divergence from the profiles of colon adenocarcinoma (COAD) and rectum adenocarcinoma (READ).

Prompted by these observations, we investigated taxonomic differences at higher resolution by transitioning to genus‐level comparisons between COAD and STAD. We first compared phylum‐level abundance using an annotated boxplot, then employed ggalign's linkage feature to map differentially abundant phyla to their associated genera (Figure 4E). The flexible layout and hierarchical grouping capabilities of ggalign enabled the representation of complex one‐to‐many relationships. This multi‐scale design revealed both broad phylum‐level trends and specific microbial contributors, showcasing ggalign's capacity to support interpretable, layered microbiome analyses.

#### 2.3.2 Case II: Pan‐Cancer Exploration of Genomic Characteristics

**Figure 5:**
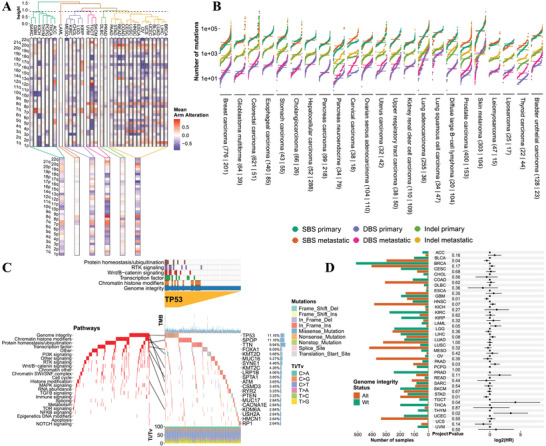
Pan‐cancer integration of genomic alterations, pathway profiles, and clinical outcomes. A) The arm‐level alterations were depicted, and hierarchical clustering using the ward D2 agglomeration method grouped the tumor types into 6 distinct clusters, with the mean arm alterations annotated. B) Comparison of SBSs, DBSs, and Indels mutation burdens between primary and metastatic tumors across cancer types. C) Oncoprints of both pathways and gene mutations in TCGA PRAD (prostate adenocarcinoma) cohort were depicted, with the lines connecting specific genetic alterations. The most significantly involved pathways in TP53‐mutated patients were annotated. D) Overall survival analysis aligned with genome instability metrics across tumor types.

To investigate genomic alterations across tumor types, we first employed ggalign to visualize arm‐level copy number alterations.^[^ ^16^, ^17^ ^]^ By applying hierarchical clustering using the Ward D2 agglomeration method, tumors were grouped into six distinct clusters based on chromosomal alteration patterns (**Figure** **5**A). While the clustering determined tumor groupings, ggalign preserved the resulting tumor type ordering and simultaneously enabled clear group‐level annotation and visualization of summarized arm‐level alterations. By maintaining the biological structure derived from clustering while supporting flexible and coherent figure composition, ggalign enhances both interpretability and communication of large‐scale genomic comparisons. We also used ggalign to replicate the figure from Thorsson et al.^[^ ^18^ ^]^ summarizing immune subtypes across pan‐cancer (Figure S6, Supporting Information), further highlighting ggalign's ability to organize complex biological classifications in a visually structured manner.

We next compared mutation burdens‐encompassing single base substitutions (SBSs), doublet base substitutions (DBSs), and insertions/deletions (Indels)‐between primary and metastatic tumors within each cancer type (Figure 5B). By leveraging ggalign's composable alignment framework along a shared y‐axis, we facilitated direct cross‐tumor comparisons without requiring manual axis adjustment or rescaling, significantly streamlining the integration of heterogeneous datasets. This capability is particularly valuable for comparative genomic analyses that span multiple tumor types with differing data ranges.

Among the findings, prostate cancer exhibited pronounced differences across all three mutation classes, with metastatic tumors displaying higher mutation burdens, particularly for SBSs and DBSs. To further delineate these genomic differences, we generated detailed mutation‐level and pathway‐level oncoprints for prostate cancer using ggalign (Figure 5C). Through its observation‐level linking mechanism, ggalign seamlessly connected gene‐level mutations with corresponding pathway alterations, enabling simultaneous visualization of mutational events and their functional consequences. This approach enhances interpretability and provides a cohesive narrative across molecular layers‐critical for identifying biologically meaningful patterns in complex data. Notably, mutations in *TP53*‐a key tumor suppressor frequently altered across malignancies‐were prevalent in both primary and, even more so, in castration‐naïve metastatic prostate tumors. By leveraging ggalign's flexible linkage capabilities, we overlaid pathway‐level summaries highlighting biological processes recurrently perturbed in *TP53*‐mutated cases, including DNA damage repair and cell cycle regulation pathways.^[^ ^19^, ^20^ ^]^ Without such structured linking across different biological layers, these relationships would be more difficult to discern, underscoring the practical value of ggalign for multi‐dimensional biological data integration.

Given that loss of genomic integrity often results in characteristic mutational signatures, we next assessed its prognostic relevance across tumor types (Figure 5D). Utilizing ggalign's flexible alignment capabilities, we synchronized forest plots of hazard ratios with corresponding genomic instability metrics. This coordinated layout enabled direct visual comparison across tumor types, revealing that tumors exhibiting higher levels of genomic instability generally corresponded to poorer prognosis, consistent with findings from other studies.^[^ ^19^, ^21^, ^22^ ^]^ By facilitating the alignment of disparate data modalities within a consistent visual framework, ggalign improves both the efficiency and clarity of complex pan‐cancer analyses.

Together, these coordinated visualizations underscore ggalign's ability to integrate multi‐modal genomic data, preserve biologically meaningful structures, and construct highly interpretable, publication‐quality layouts that facilitate the discovery of clinically relevant patterns across large, heterogeneous cohorts.

#### 2.3.3 Case III: Pan‐Cancer Single‐Cell Profiling of Tumor Microenvironment and Cell Type Distribution

**Figure 6:**
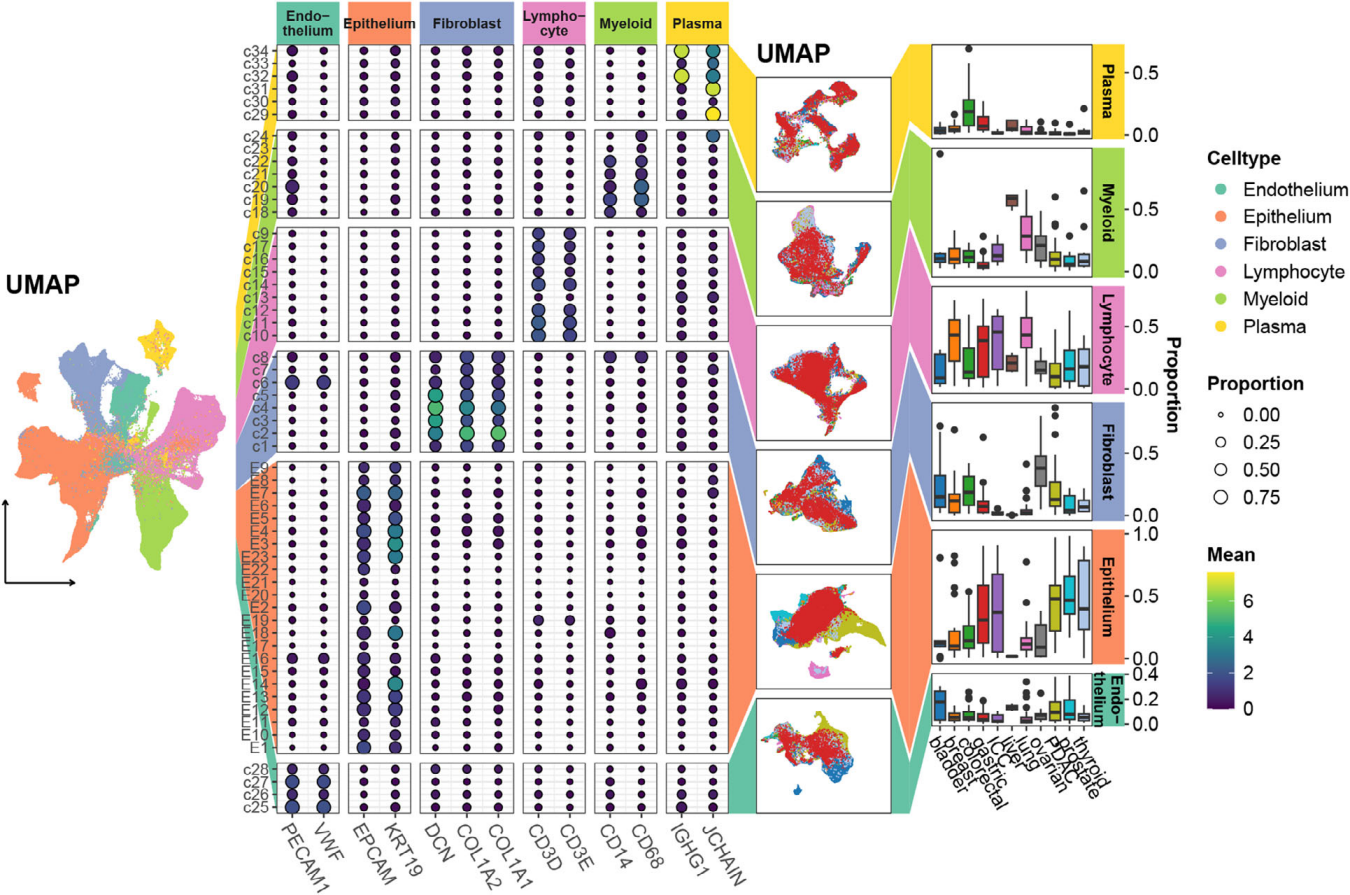
Pan‐cancer analysis of cell type distribution and abundance at single‐cell resolution. From left to right. The first plot depicted the UMAP plot of all cells. Canonical cell markers were then used to annotate the clusters, identifying 6 distinct cell types. Each cell type was visualized using a separate UMAP plot to compare its distribution across tumor types. Boxplots were used to assess the differences in cell proportions across these tumor types.

To compare the proportions of different cell types, we first clustered the single‐cell transcriptome data and visualized the results using a Uniform Manifold Approximation and Projection (UMAP) plot (**Figure** **6**). Canonical cell markers were used to annotate the clusters, identifying six distinct cell types: epithelium (*EPCAM* and *KRT19*), lymphocytes (CD3D and *CD3E*), myeloid cells (*CD14* and *CD68*), fibroblasts (*DCN*, *COL1A2*, and *COL1A1*), endothelium (*PECAM1* and *VWF*), and plasma cells (*IGHG1* and *JCHAIN*). Each of these cell types was visualized using separate UMAP plots to compare their distributions across tumor types. Additionally, boxplots were employed to assess the differences in cell proportions across tumor types. By leveraging ggalign's flexible alignment and linkage features, the UMAP plots for each cell type were visually connected to their corresponding boxplots and gene expression patterns, enabling users to simultaneously observe the spatial distributions, proportional differences, and gene expression levels of each cell type across tumor types. This integrated approach streamlines the comparison of data across different biological layers, making the visual analysis more intuitive and coherent. Ultimately, ggalign ensured that all data were presented in a unified manner, improving ease of interpretation without sacrificing the richness of the underlying biological detail. The integration of these various visualizations into a cohesive figure was greatly facilitated by ggalign, which ensured smooth coordination across components, a feature that can be applied in other contexts. The total time required for ggalign to generate Figure 6 was 9.306 s, based on input data comprising 827,322 cells.

## 3 Discussion

Data visualization is critical for the exploration, interpretation, and communication of complex biological data. As the scale and complexity of omics datasets expand, there is an increasing demand for flexible and adaptable visualization tools. In this study, we present ggalign, an R package that extends the ggplot2 ecosystem to enable the creation of unified and customizable visualizations. Offering a general‐purpose framework, ggalign overcomes limitations in existing tools, providing novel approaches for exploring multi‐omics data and enhancing scientific communication.

One of the key strengths of ggalign lies in its modular, data‐aware design, which facilitates the dynamic arrangement of visual elements tailored to a variety of biological data types. The package supports three primary layout modes‐circular, stacked, and quadrant‐based‐each optimized for specific visualization tasks such as large‐scale data representation, condition comparisons, and hierarchical structures. In contrast to tools like Marsilea^[^ ^10^ ^]^ and aplot,^[^ ^9^ ^]^ which are constrained by rigid layout structures, ggalign offers enhanced flexibility in organizing visualizations, while also accommodating diverse data relationships, including one‐to‐many and many‐to‐one connections. This combination of flexible layouts and adaptable data relationships makes ggalign particularly suited for multi‐omics research, where complex, interconnected data must be visualized effectively.

Another distinguishing feature of ggalign is its ability to reorder and group observations based on data‐driven or domain‐specific criteria. This functionality enhances the visibility of biologically meaningful patterns, especially in high‐dimensional datasets such as single‐cell transcriptomics and microbiome profiling. Our case studies demonstrate how ggalign maintains data organization across linked plots, facilitating comparisons and improving interpretability.

 ggalign also introduces a novel capability to link observations across diverse data types, enabling the visualization of intricate relationships, such as between gene mutations and pathway alterations. This feature facilitates deeper analytical insights and clearer communication of biological processes, as illustrated in both genomic and microbiome research case studies.

A current limitation of ggalign is that it supports only static visualizations within the R programming environment. While this design choice allows tight integration with the ggplot2 ecosystem and promotes reproducibility in R‐based workflows, it restricts usage in interactive or web‐based visualization frameworks and limits applicability outside of R. Extending ggalign to support interactive environments represents a potential direction for future development.

As multi‐omics research continues to evolve, ggalign provides a versatile solution for integrating and visualizing complex datasets. Its compatibility with ggplot2 ensures that users can build on familiar graphical layers, while its flexible layout options and observation‐level customization support both exploratory data analysis and detailed examination of large‐scale datasets.

## 4 Conclusion

 ggalign represents a major advancement in composable data visualization. It enables the creation of complex, multi‐layered visualizations that are both analytically meaningful and visually compelling. By linking diverse data types across multiple scales, ggalign enhances our ability to interpret complex biological datasets and will be invaluable for researchers in multi‐omics fields.

## 5 Experimental Section

### Dataset Collection

Pan‐cancer data for the intratumor microbiome was obtained from the Cancer Microbiome Atlas database (https://tcma.pratt.duke.edu/),^[^ ^23^ ^]^ while pan‐cancer single‐cell data was retrieved from Gene Expression Omnibus (https://www.ncbi.nlm.nih.gov/geo/)^[^ ^24^ ^]^ (accession: GSE210347^[^ ^25^ ^]^). Additionally, mutation data for PRAD (prostate adenocarcinoma) was downloaded from The Cancer Genome Atlas (TCGA) (https://portal.gdc.cancer.gov/).^[^ ^13^, ^14^ ^]^ Arm‐level alterations of TCGA cancer data were obtained from the supplementary file of the study by Taylor et al.^[^ ^17^ ^]^ Data for Figure 3 was sourced from the supplementary file in the study by Wang X et al.,^[^ ^26^ ^]^ and https://github.com/YuLab‐SMU/plotting‐tree‐with‐data‐using‐ggtreeExtra.^[^ ^27^ ^]^ Data for Figure S2 (Supporting Information) was downloaded from the supplementary files of the study by Dou et al.^[^ ^28^ ^]^ Figure S4 (Supporting Information) was generated using datasets included in the circlize R package. Figure S6 (Supporting Information) was based on data from the supplementary materials of Thorsson et al.,^[^ ^18^ ^]^ however, since clustering results were not provided by the authors, we annotated only representative immune signatures, resulting in a slightly modified version of the original figure.

### Single‐Cell Analysis

All analyses were conducted using the scrapper^[^ ^29^ ^]^ package. Gene expression matrices were normalized using the normalizeCounts function. To capture high cell‐to‐cell variation, 2,000 genes were selected based on their variance using the modelGeneVariances function. For UMAP, we applied Mutual Nearest Neighbors integration^[^ ^30^ ^]^ to combine cells from different datasets, facilitating their joint analysis. The batch‐corrected expression matrix of all integrated cells was then used to compute the UMAP.

### Statistical Analysis

All statistical analyses were conducted using the R stats package.^[^ ^31^ ^]^ Hierarchical clustering was performed using the *hclust()* function. The relative abundance of the intratumor microbiome was compared using the wilcoxon signed‐rank test, with *P*‐value adjusted using the Benjamini‐Hochberg (BH) method.

## Conflict of Interest

The authors declare no conflict of interest.

## Supporting information



Supporting Information

## Data Availability

The data that support the findings of this study are openly available at https://github.com/Yunuuuu/ggalign and https://github.com/Yunuuuu/ggalign‐research.
