# Supplementary material for: ggalign: Bridging the Grammar of Graphics and Biological Multilayered Complexity
Source: Adv Sci (Weinh). 2025 Sep 15;12(44):e07799. doi: 10.1002/advs.202507799 (PMC12667486; doi:10.1002/advs.202507799)
Supplement: Supplementary file 1 — Supporting Information [file ADVS-12-e07799-s001.pdf]

Supporting Information

ggalign: Bridging the Grammar of Graphics and Biological Multilayered Complexity

Yun Peng[1,2,#], Shan Jiang[1,#], Yuxuan Song[1,#,] Peng Luo[3], Jianfeng Li[4], Dehua Hu[2], Jian-Guo Zhou[5], Guangchuang Yu[6,], Tao Xu[1,*], and Shixiang Wang[2,*]*

Table S1: Feature and specification comparison

Specification	ggalign	Marsilea[10]	aplot[9]	ComplexHeatmap[7]
Reorder observations	√	√	√	Heatmap Only
Group observations into different panels	√	√	×	Heatmap Only
Clustering algorithm	K-means, hierarchical, custom	×	×	K-means, hierarchical, custom
Legends Creation	Automatic	Automatic	Automatic	Limited automatic, requires manual add
Legends Position	Anywhere; independently controllable per plot	Anywhere	Anywhere	Fixed to one of four sides
Tree	hclust, ape	hclust only	hclust, ape	hclust only
Tanglegram	√	×	×	×
3D Heatmap	√	×	×	√
Oncoplot	√	√	√	√
UpSet plot	√	√	×	√

A

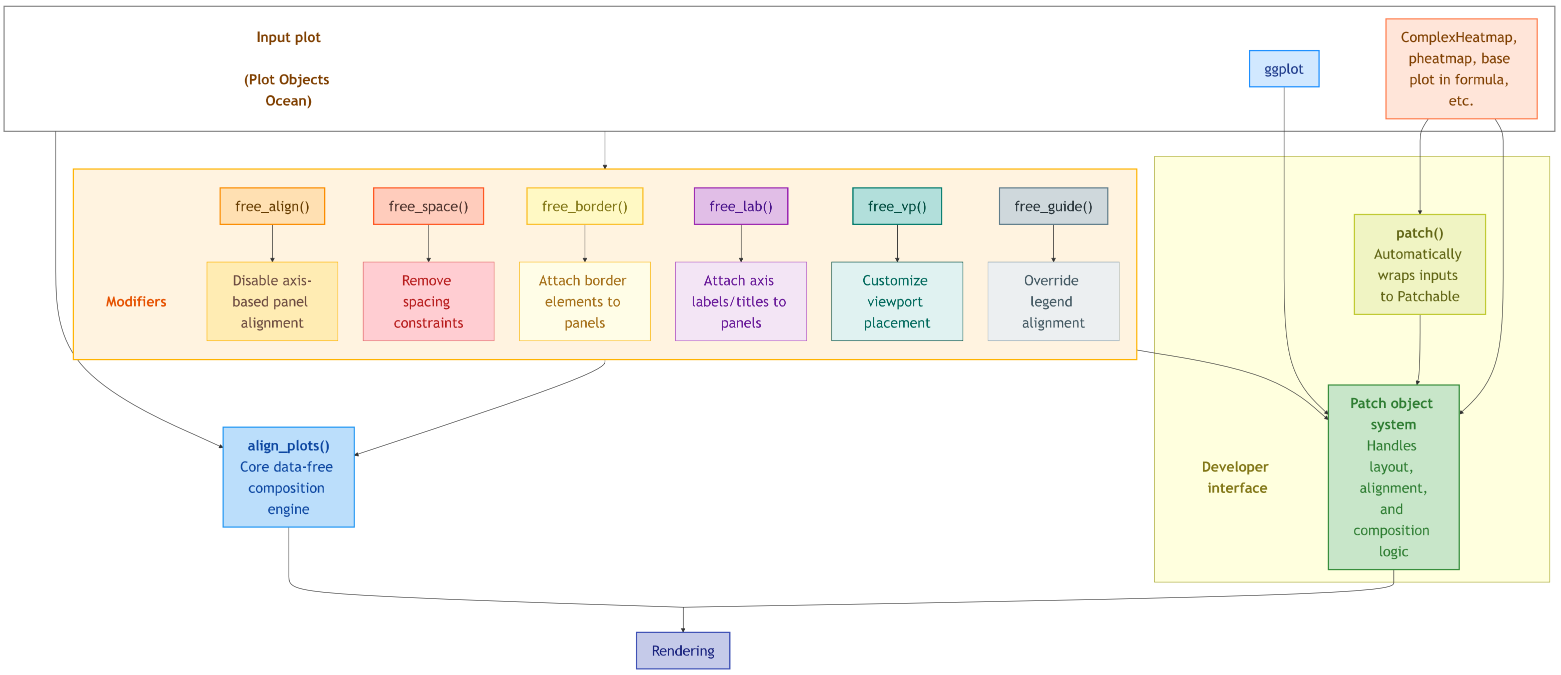

B

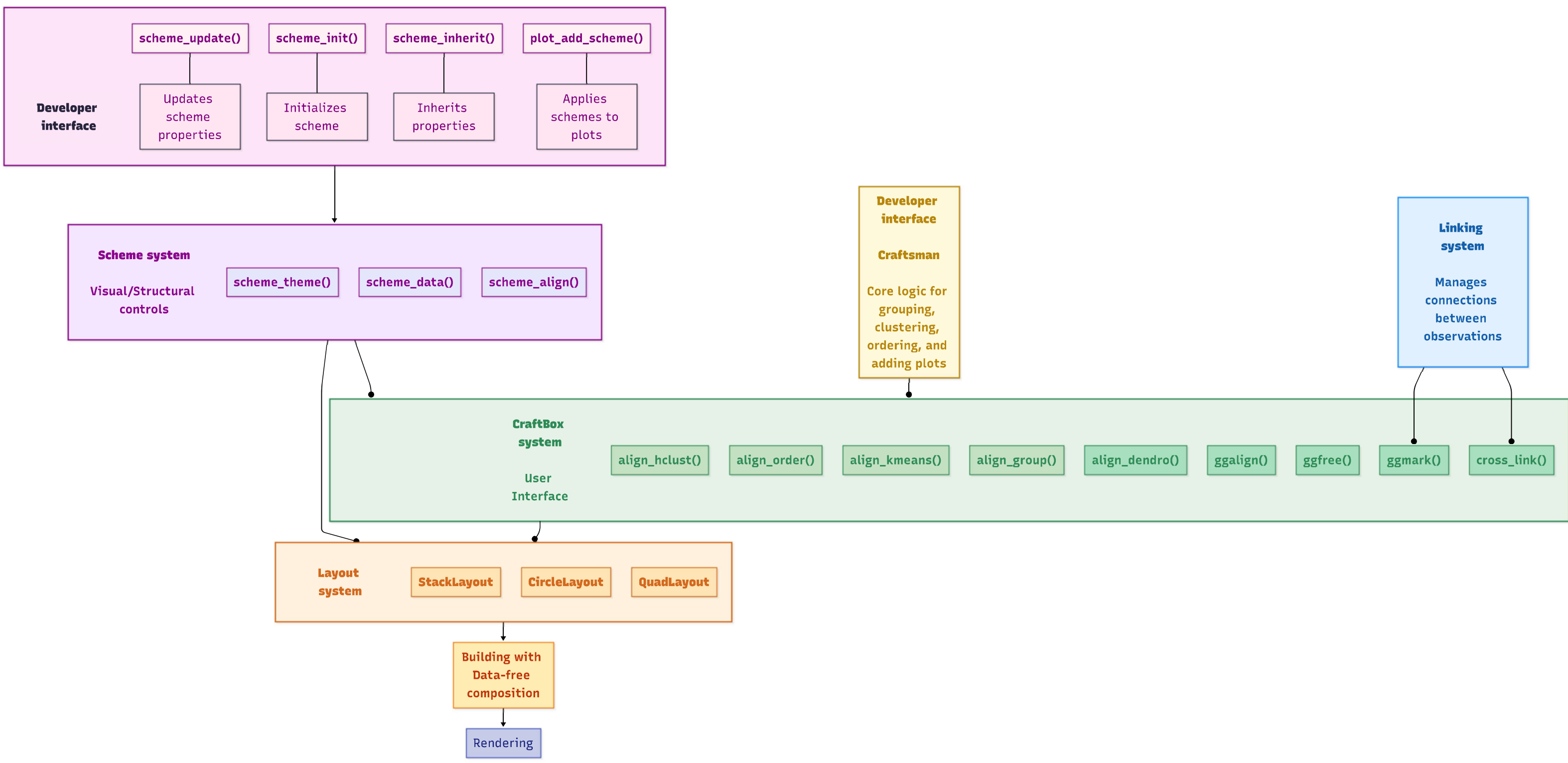

Figure S1: Conceptual overview of the object systems in ggalign. (A) The data-free composition object system. (B) The data-aware composition object system.

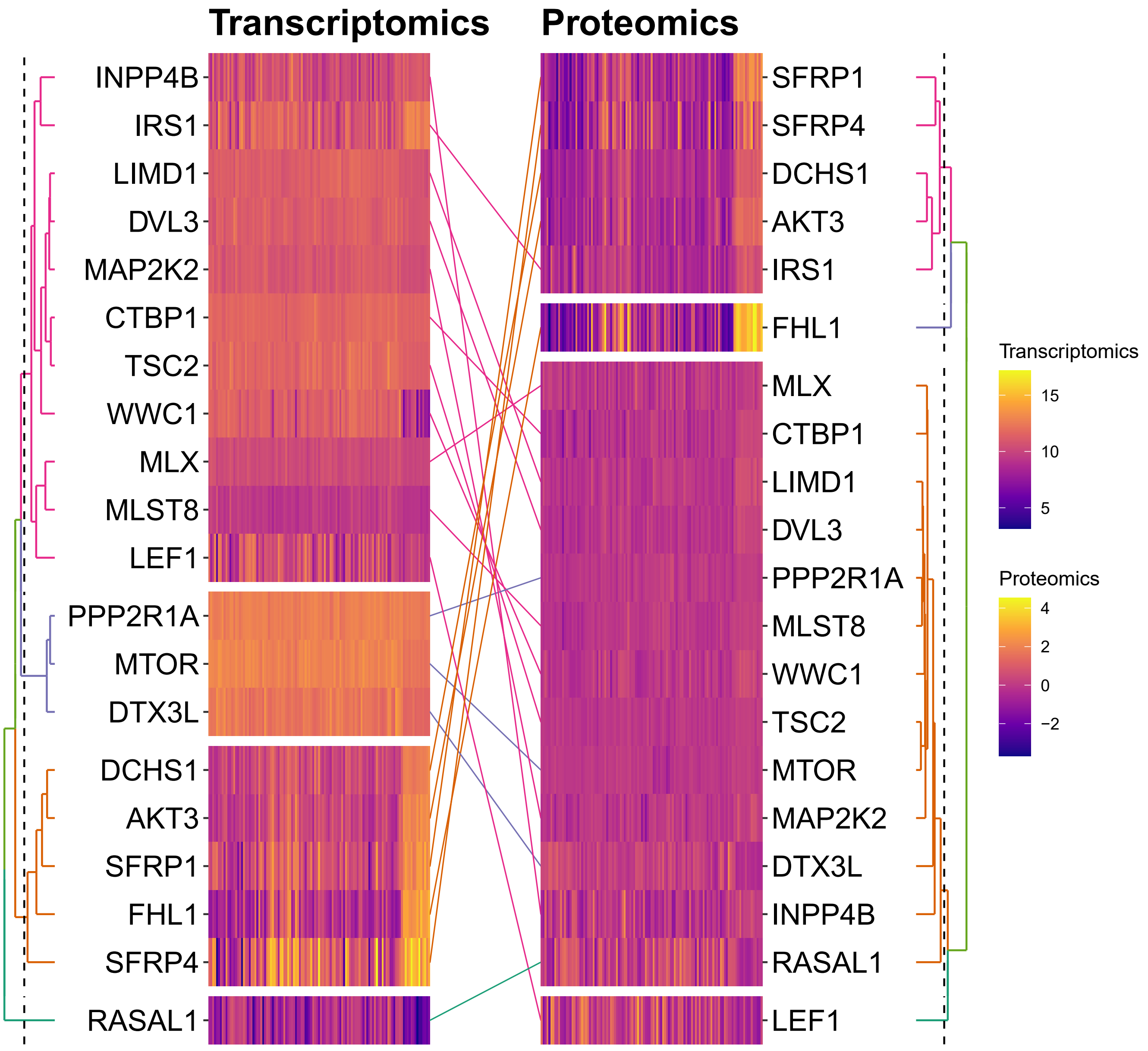

Figure S2: Coordinated and discordant expression patterns between transcriptomic and proteomic profiles in endometrial cancer.

```r
stack_alignh(data) +                       # initialize a layout
    align_hclust() +                       # reorder the data based on hierarchical clustering
    ggalign() +                            # initialize a plot
    geom_bar() +                           # add ggplot2 layer
    scale_fill_brewer(palette = "Set3") # add ggplot2 scale
```

Figure S3: Example code demonstrating data-aware operations in ggalign.

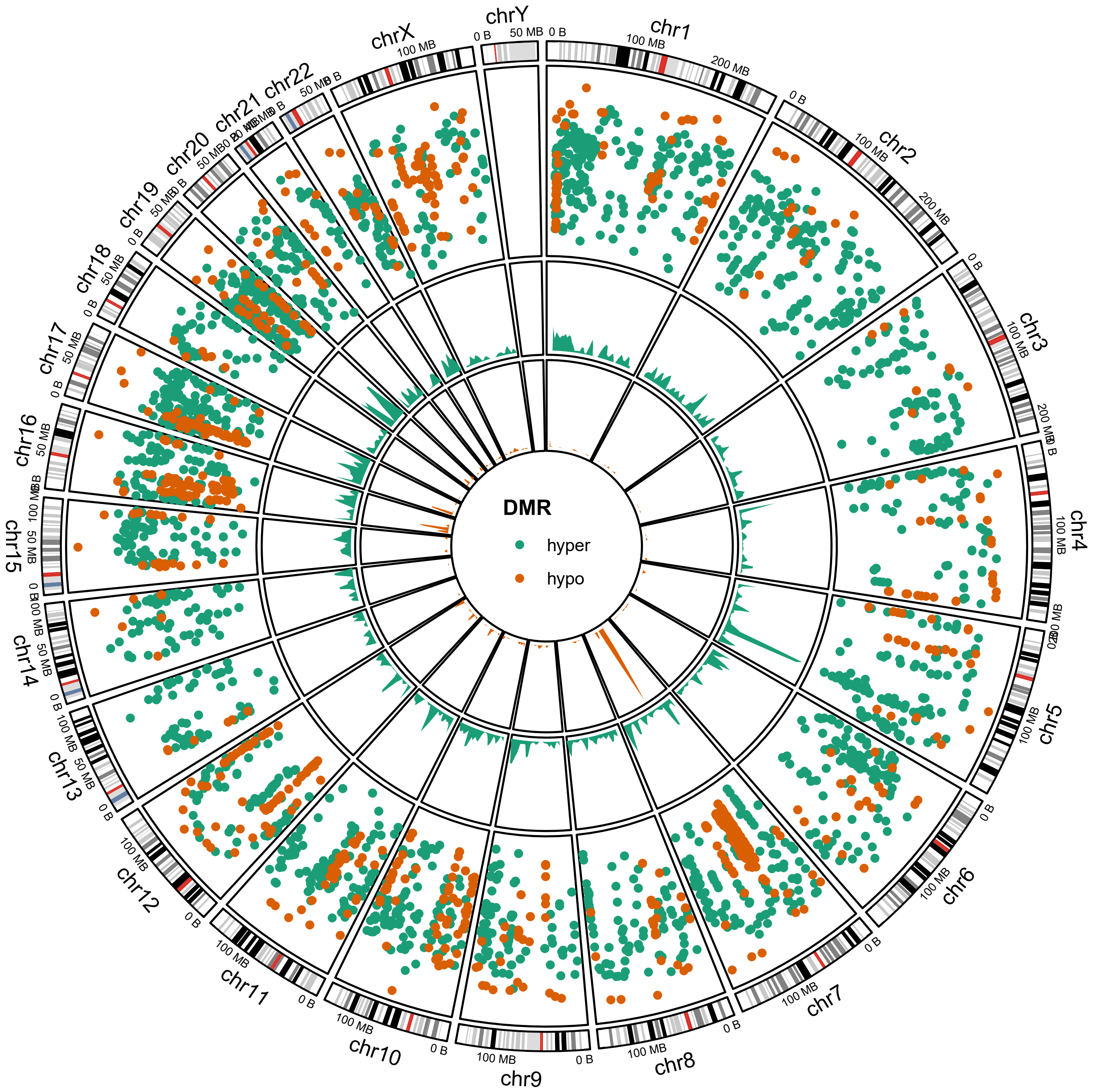

Figure S4: Genomic density and rainfall plot for differentially methylated regions.

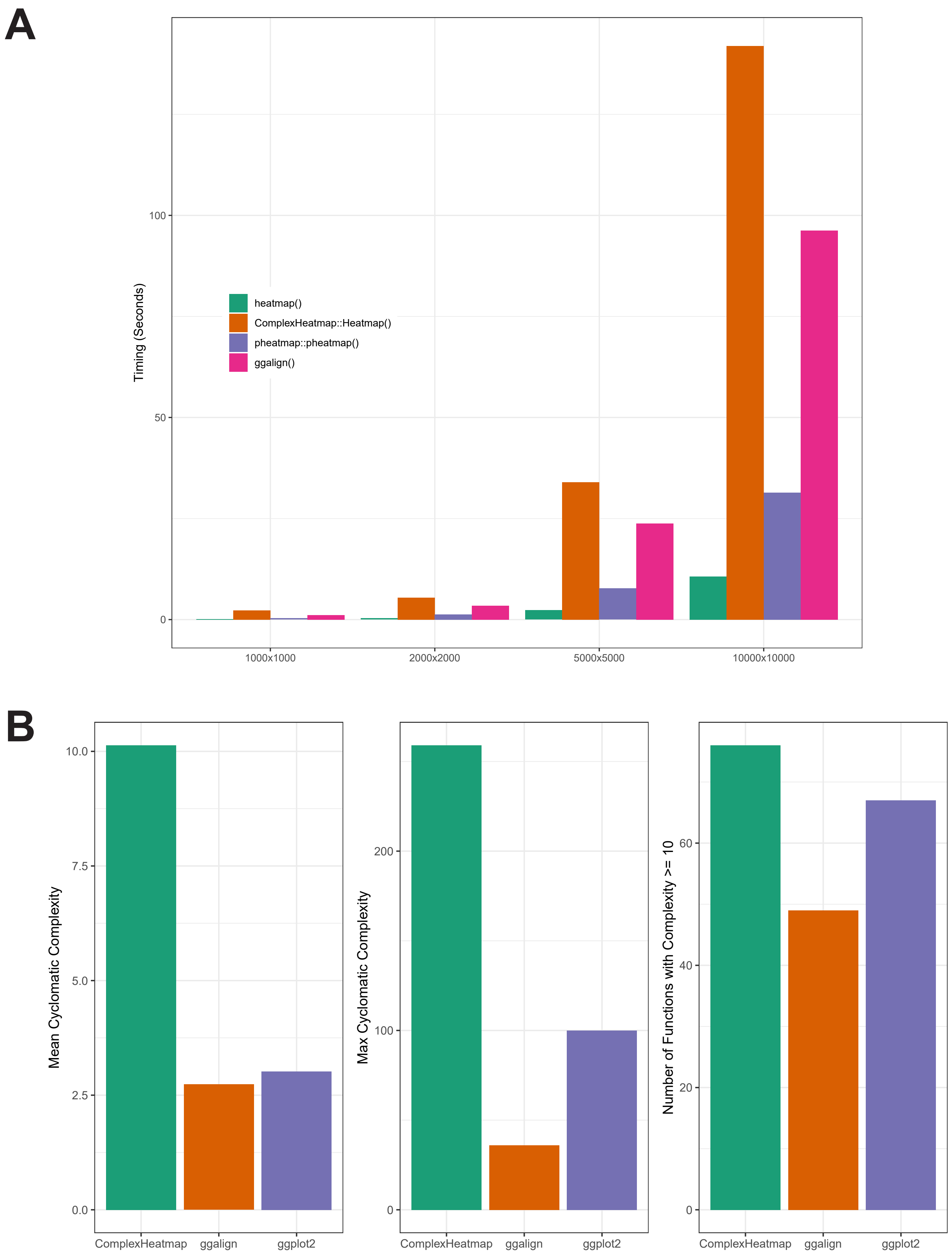

Figure S5: Performance and code complexity comparison. (A) Comparison of heatmap drawing speed across different R packages. (B) Cyclomatic complexity across different packages.

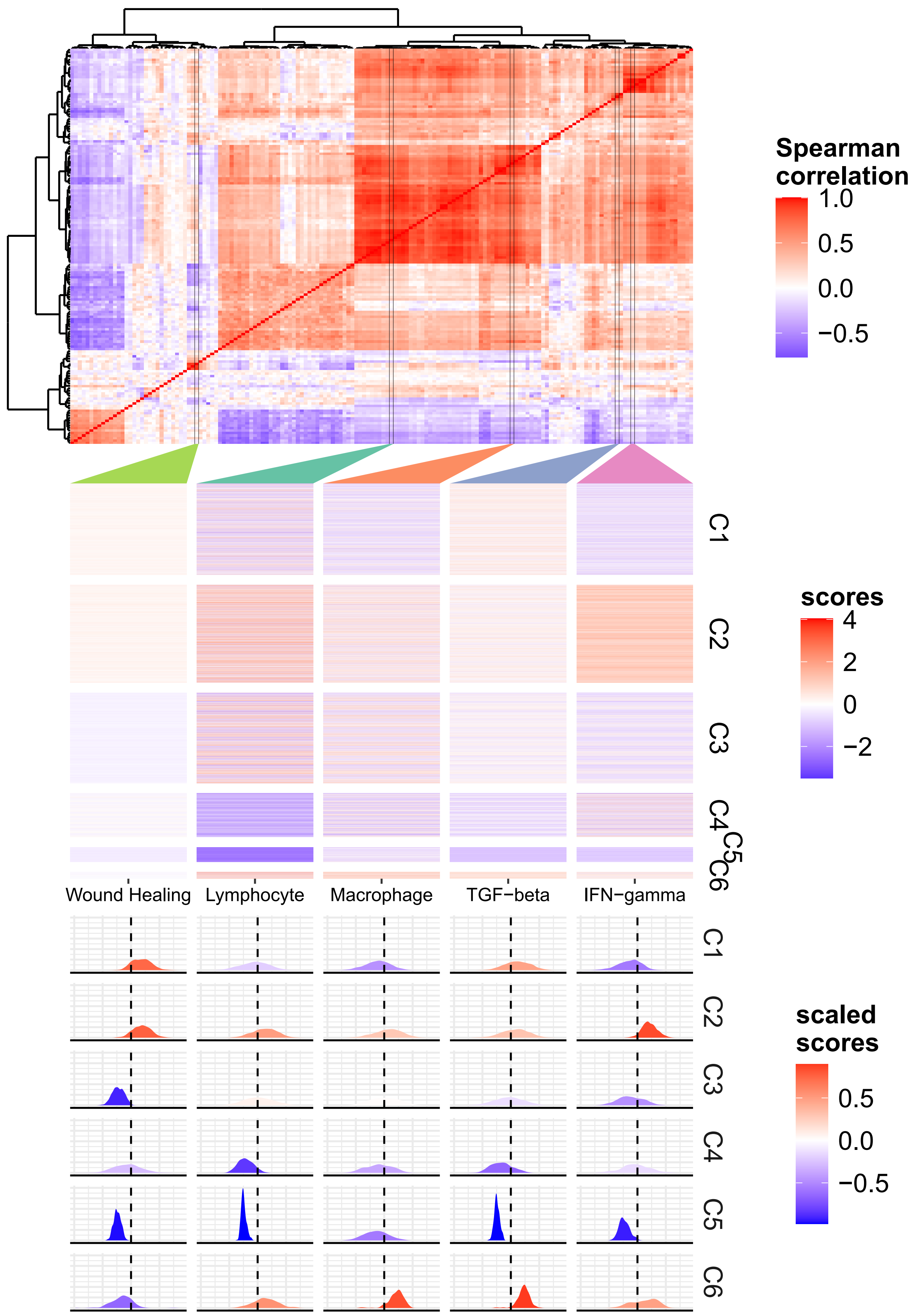

Figure S6: Pan-cancer expression signature modules and immune subtype identification. Five representative gene expression signatures (linked with colored polygons) were used to cluster TCGA tumor samples (rows), resulting in the identification of six immune subtypes, labeled C1–C6. The distribution of signature scores across the six subtypes is shown, with the dashed line indicating the median score for each subtype.
